# Pharmacokinetics of intravenous telavancin in healthy subjects with varying degrees of renal impairment

**DOI:** 10.1007/s00228-015-1847-6

**Published:** 2015-05-05

**Authors:** Philip D. Worboys, Shekman L. Wong, Steven L. Barriere

**Affiliations:** Theravance Biopharma US, Inc., 901 Gateway Boulevard, South San Francisco, CA 94080 USA; AbbVie Biotherapeutics Corp., Redwood City, CA 94063 USA

**Keywords:** Telavancin, Lipoglycopeptide, Renal impairment, Pharmacokinetics, Hydroxypropylbetadex

## Abstract

**Purpose:**

We evaluated the effect of renal impairment (RI) on the pharmacokinetics of telavancin and hydroxypropylbetadex (excipient in the telavancin drug product).

**Methods:**

Adults with normal, mild, moderate or severe RI or end-stage renal disease (ESRD) receiving haemodialysis were included in two open-label, phase I studies of single-dose telavancin at 7.5 mg/kg (study A, *n* = 29) or 10 mg/kg (study B, *n* = 43). Pharmacokinetic analysis of telavancin and hydroxypropylbetadex plasma concentration versus time was performed in these subjects.

**Results:**

The results in studies A and B were similar: telavancin systemic exposure (area under the concentration–time curve from 0 to infinity [AUC_0–∞_]) increased with RI. Telavancin half-life (*h*, mean ± SD) increased in subjects with severe RI compared with subjects with normal renal function from 6.9 ± 0.6 in study A and 6.5 ± 0.9 in study B to 14.5 ± 1.3 and 11.8 ± 6.7, respectively. Conversely, clearance (ml/h/kg, mean ± SD) decreased in subjects with severe RI compared with subjects with normal renal function from 13.7 ± 2.1 in study A and 17.0 ± 3.2 in study B to 6.18 ± 0.63 and 6.5 ± 1.5, respectively. Systemic exposures for hydroxypropylbetadex also increased with severity of RI.

**Conclusions:**

Results from two independent phase 1 studies suggest that dose adjustment of telavancin is required in subjects with varying degrees of RI.

## Introduction

Antibiotic resistance among Gram-positive bacteria, including staphylococci, has necessitated the development of new antibiotics. Vancomycin, a glycopeptide antibiotic, has been the drug of choice to treat methicillin-resistant *Staphylococcus aureus* infections. The increasing prevalence of multidrug-resistant strains, including those that are resistant to vancomycin, has resulted in the development of the lipoglycopeptide class of antibiotics, which have a lipophilic side chain linked to a glycopeptide backbone. Members of this class of antibiotics include oritavancin, dalbavancin and telavancin.

Telavancin is a bactericidal lipoglycopeptide antibiotic with activity against clinically relevant Gram-positive bacteria [1]. The antimicrobial activity of telavancin results from a dual mode of action, including inhibition of peptidoglycan synthesis and disruption of the functional integrity of the bacterial membrane [2, 3]. In the USA and Canada, intravenous (i.v.) telavancin is approved for the treatment of adult patients with complicated skin and skin structure infections caused by susceptible Gram-positive bacteria [4, 5]. In Europe, telavancin is approved for the treatment of nosocomial pneumonia, including ventilator-associated pneumonia, known or suspected to be caused by methicillin-resistant *S. aureus* when other alternatives are unsuitable [6], and for the treatment of hospital-acquired bacterial pneumonia and ventilator-associated bacterial pneumonia due to *S. aureus* when other alternatives are not suitable in the USA [5]. In healthy adult volunteers, telavancin at 7.5–15 mg/kg/day administered as an i.v. infusion displayed linear pharmacokinetics, with an elimination half-life (*t*_½_) averaging 7 h and trough plasma concentrations exceeding the minimum concentration required to inhibit the growth of 90 % of organisms for key Gram-positive pathogens [7, 8]. Following i.v. administration, telavancin is largely excreted intact in urine (82 % after a single administration of 10 mg/kg) [9], and no significant gender- or age-related differences in telavancin pharmacokinetics have been observed in healthy volunteers [8, 10]. Co-administration of telavancin with the renally excreted antibiotics aztreonam or piperacillin/tazobactam had no significant effect on the pharmacokinetics of any of the drugs [11]. The pharmacokinetic profile supports a once-daily dosage strategy for telavancin in patients with normal renal function.

It was expected that systemic exposure to telavancin would be greater in patients with reduced creatinine clearance (CL_cr_) than in those with normal CL_cr_. In order to develop dosage recommendations, we examined the pharmacokinetic profile of telavancin and the drug product excipient hydroxypropylbetadex, used in telavancin’s formulation to aid solubilisation, in subjects with various degrees of renal impairment in two phase 1 trials, Studies 103a (Theravance, Inc.) and 2403 (Astellas Pharma Europe B.V.), herein referred to as study A and study B, respectively.

## Materials and methods

### Study designs

Study A was a phase 1, open-label, single-arm, single-dose, two-centre study (Quintiles Limited, Guy’s Drug Research Centre, London, UK and MDS Pharma Services, New Orleans, LA, USA) that examined the effects of renal impairment on telavancin pharmacokinetics in subjects with varying degrees of renal impairment. Study B was a phase 1, open-label, single-dose, three-centre study in Poland (SP ZOZ Szpital Praski, Warsaw; SP Specjalistyczny Szpital, Zachodni, Grodzisk Mazowiecki; and SZP ZOZ, Wołominie, Wołomin) that investigated the effect of mild, moderate and severe renal impairment on the pharmacokinetics of telavancin compared with subjects with normal renal function.

### Ethics

The study protocols were approved by the institutional review board at each participating site, and both studies were conducted in full compliance with the principles of the International Conference on Harmonization of Technical Requirements for Registration of Pharmaceuticals for Human Use, Good Clinical Practice and the Declaration of Helsinki. Written informed consent was obtained from all subjects.

### Subjects

Subjects included men and non-pregnant women and, in study A or B, respectively, were ≥18 years or 20–79 years of age with a body mass index of 18–36 or 18.5–34 kg/m^2^. Subjects were either healthy or had mild, moderate or severe renal impairment. Subjects were excluded if they had a history of any relevant pulmonary, haematologic, hepatic, immunologic, endocrine, metabolic, rheumatic, neurologic or psychiatric disorder. Subjects with stable, adequately treated medical conditions could be enrolled provided that their medical regimen was unchanged in the 3 months before study participation and that participation did not place them at increased risk of adverse events.

Subjects were assigned to the renal impairment strata based on assessment during pre-study screening. For study A, strata assignment was based on CL_cr_ calculated using the Cockcroft-Gault (C–G) equation [12] (Table 1). For study B, assignment was based on the estimated glomerular filtration rate (eGFR) using the Modification of Diet in Renal Disease formula [13] (Table 1). Summary statistics for Study B were also prepared using the C–G equation [12] with and without normalization to body surface area. Creatinine assays were performed at each clinical site using UV absorbance assays at 512 nm to detect the yellow–red complex formed from the reaction of creatinine and picric acid under alkaline conditions.

**Table 1:** Determination of renal function by estimated pre-dose creatinine clearance and glomerular filtration rate values

Renal impairment group	Study A, CL_cr_ (mL/min)^a^	Study B, eGFR (mL/min/1.73 m^2^)^b^
Normal	>80	≥90
Mild	51–80	60 to 89
Moderate	30–50	30 to 59
Severe	<30	15 to 29
ESRD	Subjects maintained on dialysis	NA

*ESRD* end-stage renal disease, *CL*_*cr*_ creatinine clearance, *eGFR* estimated glomerular filtration rate, *NA* not applicable

^a^Cockcroft-Gault equation

^b^Modification of Diet in Renal Disease formula

In subjects with end-stage renal disease (ESRD), haemodialysis was initiated 2–4 h after study drug administration (study A only). Haemodialysis sessions were 4 h in duration, and when possible, a similar type of dialyzer at a similar blood flow rate was used across haemodialysis subjects.

### Treatments

Telavancin for injection was supplied as a sterile, lyophilized powder for i.v. injection. Each millilitre of formulated solution contained approximately 10 mg of telavancin, 100 mg of hydroxypropylbetadex (to improve solubility) and 12.5 mg mannitol as excipients. In study A, subjects with renal impairment received a single, 1-h infusion of telavancin 7.5 mg/kg on day 1. In study B, subjects received a single, 1-h infusion of telavancin 10 mg/kg on day 1. In study B, the actual dose of telavancin administered was approximately 10 % less than planned due to a residual amount of solution remaining within the infusion system.

### Pharmacokinetic assessments—sample collection

#### Plasma

Blood samples (5–6 ml) were collected in sodium heparin glass tubes and stored chilled until the plasma was harvested by centrifugation and stored at approximately –70 °C or below until transferred frozen to a central laboratory for analysis. For subjects with varying degrees of renal impairment, samples were collected in study A pre-infusion, at 1, 2, 4, 6, 8, 12, 24, 36 and 48 h after the start of infusion for all groups and also at 72 and 96 h post-infusion for subjects with severe renal impairment or ESRD. In study B, samples were collected pre-infusion and at 0.5, 1, 1.5, 2, 3, 4, 6, 8, 10, 12, 16, 24, 36, 48, 60, 72, 84 and 96 h post-dose.

#### Urine

Because the urine assay for telavancin was not optimized at the time of study A, no results were reported; the assays were subsequently conducted in study B, where samples were collected pre-infusion and at 0–6, 6–12, 12–24, 24–36, 36–48, 48–60, 60–72, 72–84 and 84–96 h after the start of infusion. Aliquots were collected and stored at −70 °C or lower.

#### ESRD

For subjects with ESRD (study A), a sample from the inflow to the dialyzer on the arterial side was collected before dialysis and at 1, 2, 3 and 4 h after the start of dialysis; dialysate fluid samples (10 ml) were collected before and at 30-min intervals during dialysis. Plasma samples were also collected at 0, 60, 120, 180 and 240 min following the start of dialysis.

### Analytical methods

In study A, plasma samples were analysed at a central laboratory (Covance Central Laboratory Services, Indianapolis, IN, USA) using a validated liquid chromatography–mass spectrometry (LC–MS) method for telavancin, which has been previously described [6]. The method was linear over the range 0.25–100 μg/ml and the lower limit of quantification (LLQ) was 0.25 μg/ml. Dialysate samples from study A were analysed at a separate central laboratory (Covance Laboratories, Madison, WI, USA) using a similar validated LC–MS method. The method was linear over the range of 0.1–25.0 μg/ml, and the LLQ in human dialysate for telavancin was 0.1 μg/ml. Study samples outside the calibration range were diluted and re-assayed. Dilution QC samples were included in batches where samples were diluted prior to analysis. The inter-day precision and accuracy of quality controls and standards during the validation was better than 13 % for all concentrations evaluated. Plasma concentrations of hydroxypropylbetadex were determined at Theravance, Inc. (South San Francisco, CA, USA) using high-performance LC (HPLC) with fluorescence analysis. Solid-phase extraction was used for sample preparation, after which hydroxypropylbetadex was complexed with a mobile phase additive (*1*-napthol). The resulting hydroxypropylbetadex sample peaks were then quantified against external standards. The standard curve range for hydroxypropylbetadex was 10–800 μg/ml, with an LLQ of 10 μg/ml.

In study B, plasma and urine concentrations of telavancin were analysed at Pharmaceutical Product Development (Richmond, VA, USA) after receipt of all samples using a validated LC–MS/MS method. The method was linear over the range 0.1–25 μg/ml for telavancin in plasma (LLQ 0.1 μg/ml) and 0.25–80 μg/ml (LLQ 0.25 μg/ml) for telavancin in urine. The inter- and intra-assay precision and accuracy of quality controls and standards during the validation was within 7.6 % in plasma and 10.2 % in urine. Plasma concentrations of hydroxypropylbetadex were determined at Pharmaceutical Research Associates (Assen, The Netherlands) by a validated HPLC fluorescence method.

### Pharmacokinetic analyses

Pharmacokinetic parameters of telavancin were determined by non-compartmental analysis using WinNonlin version 4.0.1 (study A) and version 5.3 (study B) (Pharsight, Mountain View, CA, USA); SAS version 9.1.3 (SAS Institute, Cary, NC, USA) was used to analyse the urine pharmacokinetic parameters in study B. Only samples with measureable concentrations were used for the purpose of calculating the pharmacokinetic parameters in both studies. Plasma pharmacokinetic parameters were calculated as previously described [7]. The CL reported for ESRD is the CL for the subjects (i.e., includes residual clearance and any dialysis clearance).

Clearance of telavancin by haemodialysis (CL_HD_) was calculated as CL_HD_ = Q_D_ × (C_TLV_ – D_TLV_) ÷ (*C*_TLV_), where *Q*_D_ was the dialysate flow rate, *D*_TLV_ was the telavancin concentration in the dialysate effluent and *C*_TLV_ was the telavancin concentration in the arterial blood entering the dialyzer. Dialyzers used included the PSN 210 and the 2K 2.5 Dialysate (both Gambro). The dialysate flow rate was 800 mL/min, and blood flow rates in patients ranged from 300 to 500 mL/min. Also, in study A, the cumulative amount of telavancin estimated in dialysis fluid was determined by multiplying the telavancin concentration in aliquots collected at 30-min intervals by the dialysate volume per collection period and then summing the individual hourly excretion amounts. In study B, the percentage of the dose excreted in urine was calculated as Ae_0–*t*_% = Ae_0–*t*_/dose × 100 % and Ae_0–∞_% = Ae_0–∞_/dose × 100 %. Renal CL (CL_R_) was calculated as CL_R_ = Ae ÷ AUC, with Ae and AUC both taken over the same interval and where Ae is amount of telavancin excreted over time, and AUC is area under the concentration–time curve.

### Protein binding assay

Protein binding was assayed in study A. The plasma protein binding of telavancin 50 μg/ml (spiked with 1 μg/ml of [^14^C] telavancin [Vitrax, Placentia, CA, USA]) was determined *in vitro* by analysing pre-infusion plasma samples under equilibrium dialysis. The percent of protein binding was calculated as follows:

$$ \begin{array}{l}\%\mathrm{bound}=\left(\mathrm{donor}-\mathrm{receptor}\right)\div \left(\mathrm{donor}\right)\times 100\%,\;\mathrm{and}\hfill \\ {}\%\mathrm{unbound}=\mathrm{receptor}\div \left(\mathrm{donor}\right)\times 100\%,\hfill \end{array} $$

where donor = plasma side concentration and receptor = buffer side concentration.

### Statistical analyses

Summary statistics were calculated for pharmacokinetic parameters by renal impairment group in both studies.

Concentration values within treatment and time point were evaluated for outliers using Tukey’s inter-quartile range method [14]. Source documentation was reviewed for all identified outliers for supportive evidence that would deem the values unreliable.

All outliers were included in the analyses. For all summaries and analyses, the baseline value was the last assessment taken prior to the administration of the dose of study drug.

## Results

### Subjects

In all, 29 (study A) and 43 (study B) subjects with varying degrees of renal impairment were enrolled. The mean CL_cr_ in each group of subjects with renal impairment was within the pre-defined range (Table 1), enabling comparative assessment of telavancin pharmacokinetics with respect to level of renal function. Demographic parameters for each group are presented in Table 2. One subject with mild renal impairment from study A received only a partial dose (approximately two thirds) of telavancin due to a flushing reaction on the upper body. This subject was excluded from pharmacokinetic evaluation but was included in the safety analysis.

**Table 2:** Demographics and baseline characteristics of phase I study volunteers by degree of renal impairment

	Study A/B, mean ± standard deviation				
	Normal (*n* = 6/14)	Mild (*n* = 7^a^/13)	Moderate (*n* = 6/8)	Severe (*n* = 4/8)	ESRD (*n* = 6/0)
Age (years)	51 ± 9/53 ± 6	59 ± 15/57 ± 5	70 ± 9/65 ± 6	58 ± 12/67 ± 9	47 ± 6/NA
Weight (kg)	79 ± 10/70 ± 12	76 ± 15/70 ± 14	69 ± 11/77 ± 13	80 ± 17/73 ± 15	81 ± 23//NA
Height (cm)	175 ± 6/164 ± 7	169 ± 7/165 ± 13	168 ± 11/162 ± 8	173 ± 15/163 ± 6	172 ± 7/NA
BMI (kg/m^2^)	26 ± 2/26 ± 3	26 ± 4/25 ± 3	25 ± 4/29 ± 4	27 ± 2/28 ± 5	27 ± 7/NA
CL_cr_ (mL/min)/eGFR (mL/min/1.73 m^2^)^b^	94 ± 11/110 ± 20	67 ± 9/ 81 ± 6	40 ± 7/48 ± 7.5	22 ± 7/ 21 ± 5	10 ± 4/NA

*BMI* body mass index, *CL*_*cr*_ creatinine clearance, *ESRD* end-stage renal disease, *NA* not applicable, *SD* standard deviation

^a^One subject was excluded from pharmacokinetic evaluation due to receipt of an incomplete dose of telavancin but was included in the safety/demographic population

^b^See Table 1 for definitions of CL_cr_ (mL/min) from study A and eGFR (mL/min/1.73 m^2^ ) from study B

### Pharmacokinetics

#### Telavancin

**Fig. 1:**
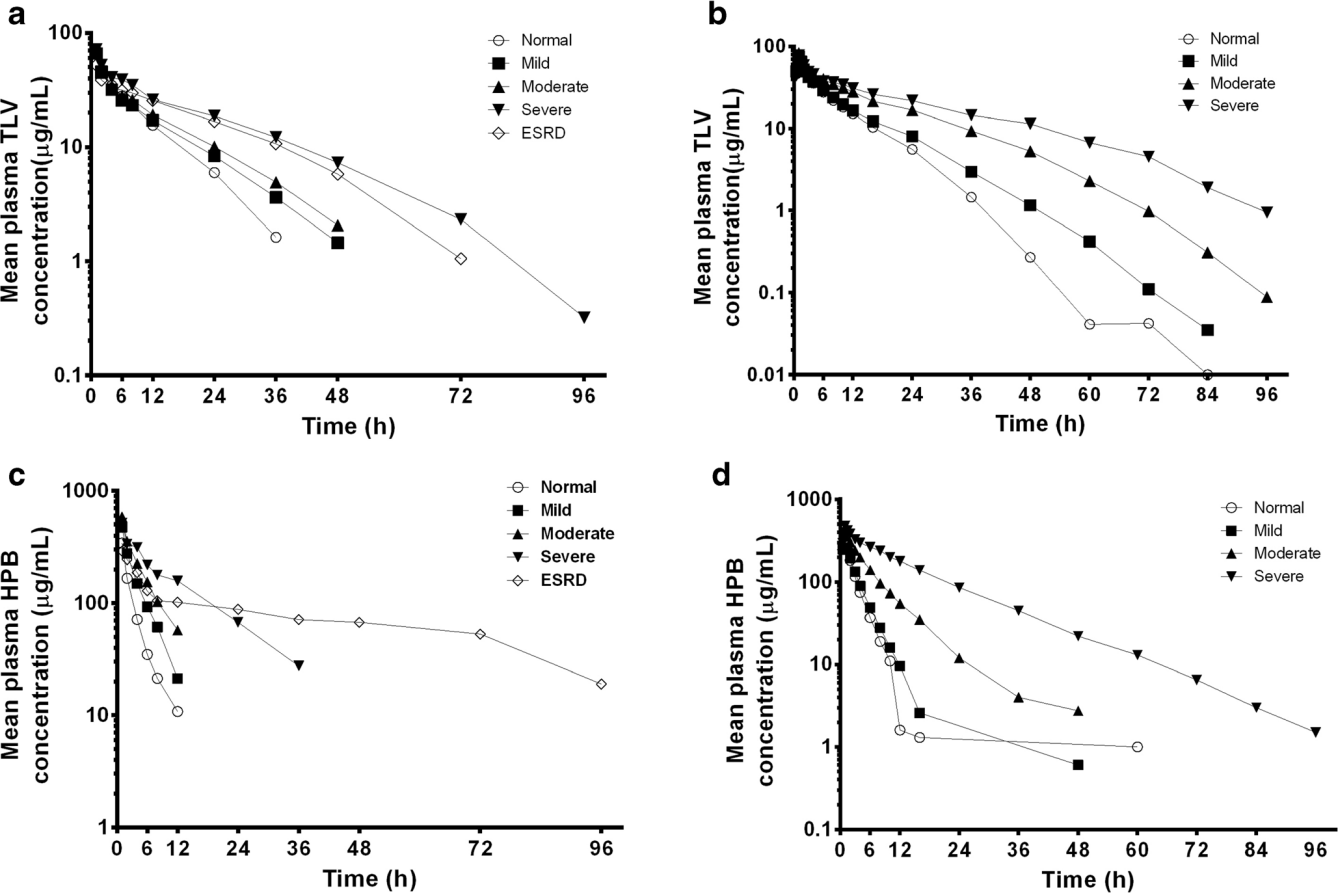
Mean plasma telavancin (*TLV*; **a**, **b**) and hydroxypropylbetadex (*HPB*; **c**, **d**) concentration–time profiles in subjects from studies A and B, respectively, with normal renal function and those with mild impairment, moderate impairment, severe impairment, or end-stage renal disease (*ESRD*)

**Fig. 2:**
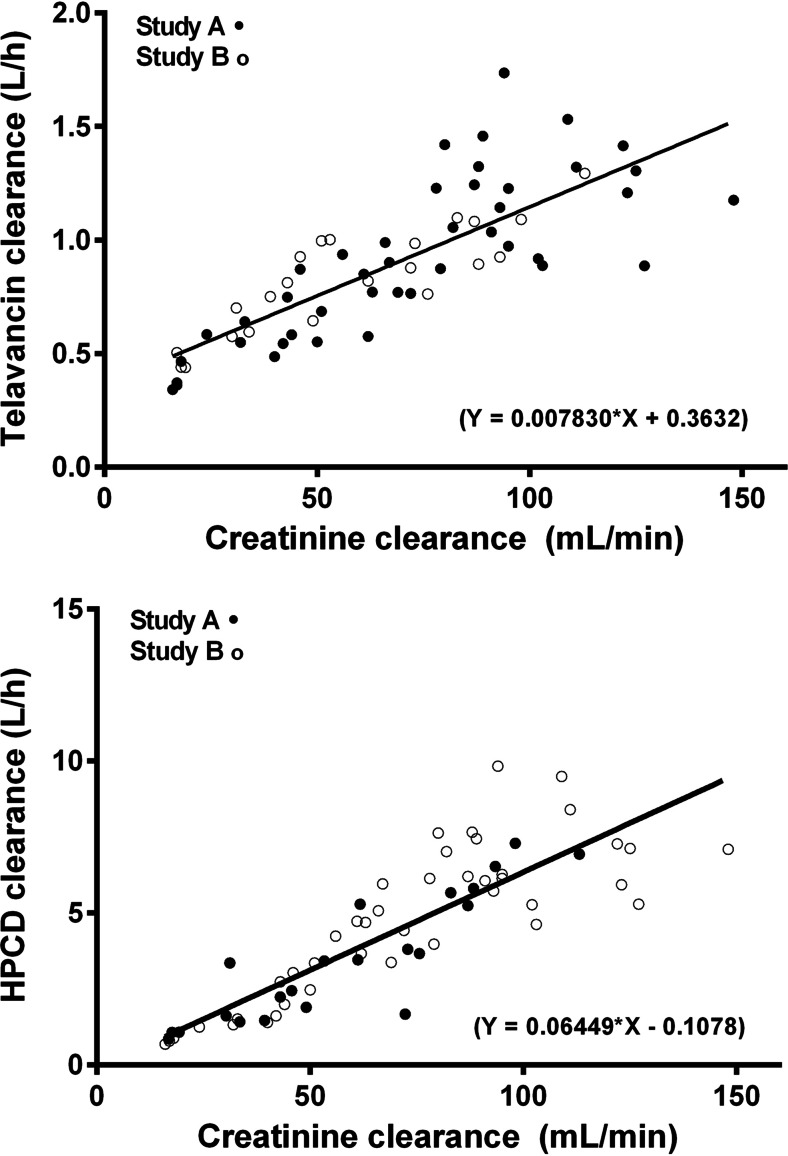
Correlation plots of creatinine clearance versus **a** telavancin and **b** hydroxypropylbetadex total body clearance. CL_cr_ was calculated using the Cockcroft–Gault equation without normalization for body surface area

For both studies, following a single, 1-hour i.v. infusion of telavancin 7.5 mg/kg (study A) or 10 mg/kg (study B), plasma telavancin concentrations declined in an apparent bi-exponential manner, with a slower decline in subjects with greater degrees of renal impairment (Fig. 1a, b; study A and B, respectively). The distribution of telavancin, as measured by mean maximum concentration and volume at steady state (*V*_ss_), was similar in healthy subjects and those with varying degrees of renal impairment (Table 3). In subjects with severe renal impairment or ESRD, mean plasma telavancin CL was reduced by more than 50 %, and *t*_1/2_ almost doubled in both studies. A strong relationship was found between plasma CL of telavancin and CL_cr_ over the entire range of renal function (Fig. 2a): *r*^*2*^ = 0.76 in study A and *r*^*2*^ = 0.62 in study B. Based on comparison of AUC from time zero to infinity (AUC_0–∞_) estimates in healthy subjects, systemic exposure to telavancin in study A/study B was increased by 13/12, 29/74 and 118/160 % in subjects with mild, moderate and severe renal impairment, respectively. The corresponding increase in AUC_0–∞_ in the ESRD group in study A was 79 %. In subjects who underwent haemodialysis on the same day as administration of 7.5 mg/kg of telavancin, approximately 6 % of the total administered dose was removed by dialysis session. The dialysis clearance was estimated to be 4.5 ml/min (271 ml/h; approximately 25 % of clearance in normal subjects).

**Table 3:** Mean (±standard deviation) non-compartmental pharmacokinetic parameters of telavancin and the excipient hydroxypropylbetadex in subjects with varying degrees of renal function (see Table 1 for definitions CL_cr_ (ml/min) from study A and eGFR (ml/min/1.73 m^2^) from study B)

Parameter	Study A/study B, mean ± standard deviation				
	Normal (*n* = 6/14)	Mild (*n* = 6/13)	Moderate (*n* = 6/8)	Severe (*n* = 4/8)	ESRD^a^ (*n* = 6/0)
Telavancin					
Protein binding (%)^a^	86.5 ± 1.3	87.5 ± 1.0	87.8 ± 1.1	86.7 ± 1.2	87.6 ± 1.0/0
*t*_½_ (h)	6.9 ± 0.6/6.5 ± 0.9	9.6 ± 2.9/7.8 ± 2.0	10.6 ± 2.4/8.1 ± 1.5	14.5 ± 1.3/11.8 ± 6.7	11.8 ± 2.8/0
*C*_max_ (μg/mL)	70.6 ± 11.2/76.7 ± 8.6	65.9 ± 2.7/74.2 ± 7.3	65.8 ± 12.1/78.0 ± 4.5	71.8 ± 7.1/83.2 ± 13.1	52.1 ± 10.1/0
AUC_0–∞_ (μg∙h/mL)	560 ± 93/539 ± 99	633 ± 101/606 ± 139	721 ± 200/936 ± 182	1220 ± 120/1397 ± 297	1010 ± 341/0
CL (mL/h/kg)	13.7 ± 2.1/17.0 ± 3.2	12.1 ± 1.9/15.2 ± 4.6	11.1 ± 3.3/9.5 ± 2.0	6.18 ± 0.63/6.5 ± 1.5	8.18 ± 2.65/0
*V*_ss_ (mL/kg)	131 ± 16/155.4 ± 30.2	157 ± 19/159.5 ± 34.5	156 ± 24/157.8 ± 41.7	136 ± 10/ 141.9 ± 17.7	157 ± 27/0
Hydroxypropylbetadex					
*t*_½_ (h)	3.02 ± 1.56/2.15 ± 0.35	4.96 ± 6.22/2.47 ± 0.55	4.18 ± 1.27/5.09 ± 2.07	9.84 ± 3.62/12.2 ± 2.8	37.2 ± 8.72/0
*C*_max_ (μg/mL)	344 ± 61/386 ± 51	476 ± 83/367 ± 37	565 ± 172/453 ± 58	517 ± 36/479 ± 57	290 ± 59/0
AUC_0–∞_ (μg∙h/mL)	946 ± 45/971 ± 199	1880 ± 1050/1083 ± 275	2720 ± 1190/2512 ± 921	5440 ± 1460/6385 ± 1713	8510 ± 2470/0
CL (mL/h/kg)	79.5 ± 3.8/94.5 ± 19.0	49.2 ± 22.7/85.1 ± 25.3	32.5 ± 14.2/37.2 ± 11.0	14.5 ± 3.3/14.6 ± 4.5	9.8 ± 4.3/0
*V*_ss_ (mL/kg)	263 ± 101/277.7 ± 148.2	182 ± 51/248.6 ± 61.8	160 ± 71/228.2 ± 56.1	183 ± 23/227.8 ± 32.7	473 ± 70/0

The CL reported for ESRD is the CL for the subjects (i.e., includes residual clearance and any dialysis clearance)

*AUC* area under concentration-time curve, *ESRD* end-stage renal disease, *C*_*max*_ maximum plasma concentration, *CL* clearance, *CL*_*cr*_ creatinine clearance, *t*_*½*_ terminal elimination half-life, *V*_*ss*_ apparent volume of distribution at steady state

^a^Study A only

Plasma protein binding of telavancin *in vitro* was determined for all subjects who provided a pre-dose plasma sample in study A only. There were no appreciable differences among renal impairment groups in the degree of telavancin protein binding: the average binding of telavancin to plasma proteins was approximately 87 % in all groups (Table 3).

Urinalysis results from study B (Table 4) show that the excretion of telavancin (Ae_0–t_, normalized to a telavancin 10 mg/kg dose) decreased with declining renal function from a mean (± standard deviation) of 436 ± 91 mg in subjects with normal renal function to 207 ± 62 mg in subjects with severe renal impairment, corresponding to 62.8 and 28.1 % of the dose, respectively. All other values (Ae_0–∞_, Ae_0–∞_%) reflected the same trend. CL_R_ decreased with renal function from 10.2 ± 1.8 to 1.8 ± 0.6 ml/h/kg, becoming significant in subjects with moderate or severe renal impairment (*P* < 0.0001).

**Table 4:** Summary statistics of renal-related urinary pharmacokinetic parameters by renal function category (study B)

Parameter	Mean ± standard deviation			
	Normal renal function	Mild renal impairment	Moderate renal impairment	Severe renal impairment
Telavancin				
Ae_0-t_ (mg)^a^	436 ± 91	397 ± 100	368 ± 91	207 ± 62
Ae_0-t_ % (%)	62.8 ± 9.0	58.2 ± 11.5	48.4 ± 8.7	28.0 ± 4.3
Ae_0–∞_ (mg)^a^	435 ± 91	396 ± 100	375 ± 96	208 ± 60
Ae_0–∞_% (%)	62.7 ± 9.1	58.2 ± 11.6	48.4 ± 9.3	28.2 ± 3.9
CL_R_ (mL/h/kg)	10.5 ± 1.9	8.8 ± 3.3	4.7 ± 1.6	1.9 ± 0.7
Hydroxypropylbetadex				
Ae_0-t_, 10 mg (mg)^a^	4801 ± 1390	4292 ± 1449	5190 ± 709	4879 ± 1112
Ae_0-t_ % (%)	68.8 ± 15.9	63.6 ± 20.5	69.0 ± 8.6	65.7 ± 11.4
Ae_0–∞_ (mg)^a^	4786 ± 1487	4274 ± 1470	5257 ± 773	4969 ± 1070
Ae_0–∞_ (%)	68.5 ± 17.5	63.4 ± 21.1	68.7 ± 9.3	67.1 ± 12.0
CL_R_ (mL/h/kg)	62.9 ± 15.4	51.4 ± 19.4	26.5 ± 7.6	10.0 ± 4.1

^a^Normalized to a telavancin dose of 10 mg/kg

*Ae*_*0–t*_ amount of telavancin excreted in the urine up to the last quantifiable sample, *Ae*_*0–∞*_ amount of telavancin excreted in the urine extrapolated to infinity, *CL*_*R*_ renal clearance

#### Hydroxypropylbetadex

In each renal impairment group, the concentration of hydroxypropylbetadex decreased over time in a log-linear manner, indicating first-order disposition processes (Fig. 1c, d; study A and B, respectively). The CL decreased with declining renal function (Table 3), and a linear relationship was found between plasma clearance of hydroxypropylbetadex and CL_cr_ (Fig. 2b). Plasma clearance of hydroxypropylbetadex was progressively lower in the groups of subjects with increasing degrees of renal impairment and decreased by up to 88 % in study A subjects with ESRD (84 % in study B). Thus, after a single, 1-hour i.v. infusion, plasma levels of hydroxypropylbetadex remained measurable for the longest period in the groups with the poorest kidney function: hydroxypropylbetadex *t*_1/2_ was about three (study A) to six (study B) times as high in subjects with severe renal impairment and 12-fold higher in those with ESRD (study A only) relative to those with normal renal function (Table 3).

A plot of total body CL versus CL_cr_ shows an excellent correlation between the two parameters, with complete overlap of the results of the two studies: telavancin (Fig. 2a) and hydroxypropylbetadex (Fig. 2b).

### Safety and tolerability

A single i.v. infusion of telavancin 7.5 mg/kg (study A) or 10 mg/kg (study B) was well tolerated in subjects with normal renal function as well as in those with varying degrees of renal impairment. Treatment-emergent adverse events (TEAEs) in study A and B, respectively, were reported by 17/29 (59 %) and 7/43 (16.3 %) of the subjects, including 5/6 (83 %) and 0/14 (0 %) with normal renal function, 4/7 (57 %) and 1/13 (7.7 %) with mild renal impairment, 3/6 (50 %) and 2/8 (25 %) with moderate renal impairment and 3/4 (75 %) and 4/8 (50 %) with severe impairment as well as 2/6 (33 %) subjects with ESRD (study A only). The most common TEAEs reported in study A were foamy urine (*n* = 6), dysgeusia (*n* = 5), nausea (*n* = 4), headache (*n* = 3), dizziness (*n* = 2), abdominal distension (*n* = 2) and somnolence (*n* = 2). One subject with mild renal impairment experienced flushing of the upper body (consistent with ‘red man’ syndrome), resulting in discontinuation of the infusion after two thirds of the dose had been administered. This subject was treated with i.v. chlorpheniramine and recovered fully.

No clinically significant changes in haematology, blood chemistry, urinalysis, vital signs, physical signs or electrocardiogram (ECG) were detected following telavancin administration. Similarly, no clinically significant abnormalities were detected in ten subjects with various degrees of renal impairment who underwent audiology assessments. In study B, the most frequently reported event was ECG QT prolongation as observed in three subjects (7.0 %), followed by increased blood lactate dehydrogenase in two subjects (4.7 %); these were mild and transient. Prolongation in ECG QT interval was reported in two subjects with severe renal impairment and one subject with moderate renal impairment; all cases were transient and reported as abnormal and clinically significant by the investigator. No serious adverse events were reported.

## Discussion

There is an increase in AUC and *t*_1/2_ for telavancin and a decrease in CL with increasing renal impairment, but no substantive change in maximum concentration after a single-dose administration. Additionally, the data suggest that, while the renal route is clearly important, other routes of elimination may also be involved.

In study B, urine was collected for 96 h, which is more than sufficient for complete excretion, based on the estimates of *t*_½_, even in severe renal impairment. Despite this, only 28 % of the administered dose was recovered unchanged, suggesting that renal elimination of unchanged telavancin may not be the primary route of elimination in patients with moderate to severe renal impairment. Following i.v. administration, telavancin is largely excreted intact in urine (82 % after a single administration of 10 mg/kg) [9], with the remaining dose being eliminated as several hydroxylated metabolites. In subjects with increasing renal impairment, the renal contribution to CL will be reduced in favour of the metabolic component, resulting in a reduced percentage of telavancin being recovered unchanged.

Based on previous data from subjects with normal renal function, the *t*_½_ of hydroxypropylbetadex was 2.5 ± 0.84 h (data on file) [15]. In previously studied patients with severe renal impairment, the *t*_½_ of hydroxypropylbetadex was 15.6 ± 6.0 h, and total CL was 0.67 ± 0.2 mL/h/kg (data on file, referenced in [15]), values that are consistent with the results of our study. There was no clear change in *V*_ss_ of hydroxypropylbetadex with worsening renal function, although total body CL increased in proportion to decreases in eGFR, indicating that renal impairment reduces hydroxypropylbetadex CL, resulting in an increase in AUC. However, given the dosage adjustment in patients with moderate (CL_cr_ 30–50 mL/min) and severe (10– < 30 mL/min) renal impairment, hydroxypropylbetadex exposure is expected to be reduced.

In study A, approximately 6 % of the telavancin dose was eliminated during a 4-h haemodialysis session, but the calculated haemodialysis clearance is approximately 25 % of total body CL. This observation suggests that a longer duration of dialysis might clear a larger proportion of drug dose.

Plasma concentrations of hydroxypropylbetadex were also higher in subjects with renal impairment in both studies as well as in haemodialysis patients (study A only). However, dialysis is known to remove hydroxypropylbetadex [15]. The differential effect of renal impairment on telavancin exposure versus hydroxypropylbetadex exposure may be due to a lack of metabolic clearance.

To conclude, dose adjustment of telavancin in patients with renal impairment is supported by the findings of two independent phase 1 studies in subjects with varying degrees of renal impairment. Telavancin 10 mg/kg every 24 h is recommended for patients with CL_cr_ >50 mL/min, 7.5 mg/kg every 24 h for patients with CL_cr_ 30–50 mL/min and 10 mg/kg every 48 h for those with CL_cr_ 10–30 mL/min. Results from two independent phase 1 studies suggest that dose adjustment of telavancin is required in subjects with varying degrees of RI.
